# Sex Differences in the Sustained Effects of Ketamine on Resilience to Chronic Stress

**DOI:** 10.3389/fnbeh.2020.581360

**Published:** 2020-10-20

**Authors:** Tracy Okine, Ryan Shepard, Elise Lemanski, Laurence Coutellier

**Affiliations:** ^1^Department of Psychology, The Ohio State University, Columbus, OH, United States; ^2^Department of Neuroscience, The Ohio State University, Columbus, OH, United States

**Keywords:** ketamine, stress resilience and vulnerability, prefrontal cortex, sex differences, chronic stress, parvalbumin

## Abstract

Exposure to stress is recognized to be a triggering factor in several mood disorders, including depression and anxiety. There is very little understanding of why female subjects have a significantly higher risk for these conditions than males. Recent findings in male rodents indicated that prophylactic ketamine can prevent the development of a stress-induced depressive-like phenotype, providing a pharmacological tool to study the mechanisms underlying stress resilience. Unfortunately, none of these studies incorporated female subjects, nor did they provide a mechanistic understanding of the effects of ketamine on stress resilience. Our previous work identified the prefrontal glutamatergic and parvalbumin (PV) systems as potential molecular mechanisms underlying sex differences in susceptibility to stress-induced emotional deregulations. To further address this point, we treated male and female mice with a single dose of ketamine before exposure to a chronic stress paradigm to determine whether stress-resilience induced by a pre-exposure to ketamine is similar in males and females and whether modulation of the prefrontal glutamatergic and PV systems by ketamine is associated with these behavioral effects. Ketamine prevented chronic stress-induced changes in behaviors in males, which was associated with a reduction in expression of PV and the NMDA receptor NR1 subunit. Ketamine did not protect females against the effects of chronic stress and did not change significantly prefrontal gene expression. Our data highlight fundamental sex differences in the sustained effects of ketamine. They also further implicate prefrontal glutamatergic transmission and PV in resilience to chronic stress.

## Introduction

Depression and anxiety are mood disorders characterized by an inherent sex bias. Not only are women at increased risk for developing these conditions (Kendler, 1998; Kessler, 2003), but they also often display more severe symptoms and higher prevalence of experiencing anxiety as a comorbid symptom to depression (de Graaf et al., 2002; Schoevers et al., 2003). In many instances these mood disorders are triggered by exposure to a single or succession of stressful events (Gold and Chrousos, 2002; Duman and Monteggia, 2006), suggesting that women might be more vulnerable to the effects of stress while men might display some resilience. Studies have focused on sex differences in the neuroendocrine response to stress (Bangasser and Valentino, 2014) or in gene expression in particular brain regions (Hodes et al., 2015) to explain this increased vulnerability in women. Unfortunately, other neural circuits and molecular mechanisms that are known to be major contributors to mood disorders have been largely ignored, limiting our understanding of sex-specific vulnerability to stress-related mood disorders. For instance, both clinical and preclinical studies highlighted deregulations of the prefrontal cortex (PFC) in mood disorders (Baxter et al., 1989; Mayberg et al., 2005; Covington et al., 2010). Specifically, reduced activity of the PFC is a hallmark of anxiety disorders and depression (Martinot et al., 1990; Shin et al., 2001; Thompson et al., 2015). This brain region is of particular importance since, in preclinical studies, the PFC of females was found to be especially sensitive to stress (Garrett and Wellman, 2009; Shansky et al., 2010).

Recent work supports the idea that the female PFC might be particularly vulnerable to stress and that its level of activity underlies an increased risk for stress-related mood disorders. The activity of the PFC is regulated by connections between glutamatergic excitatory neurons and inhibitory GABAergic interneurons. The female prefrontal GABAergic system, and particularly parvalbumin (PV) interneurons, appears to be particularly sensitive to chronic stress in mice (Shepard et al., 2016; Page et al., 2019). PV is a calcium-binding protein expressed in a subpopulation of cortical GABAergic interneurons characterized by fast-spiking activity. In females, chronic stress leads to increased activity of prefrontal PV interneurons, potentially due to a stress-induced increase in glutamatergic transmission onto those interneurons (Shepard and Coutellier, 2018). These findings reveal sex-specific changes in glutamatergic and GABAergic transmission in the PFC that could underlie the increased vulnerability of females to stress-related mood disorders.

Here, we used a pharmacological approach in a mouse model of chronic stress-related disorders to further test this idea. Ketamine, a nonselective NMDA receptor antagonist, has been shown to have rapid and sustained antidepressant actions (Berman et al., 2000; Zarate et al., 2006). Preclinical models have been widely used to decipher the molecular mechanisms of action of ketamine after various environmental exposures known to induce depressive-like behaviors in rodents (i.e., severe acute stress; chronic stress; social isolation—Maeng et al., 2008; Koike et al., 2011; Li et al., 2011; Sarkar and Kabbaj, 2016). Ketamine exerts its antidepressant effects by acting on NMDA receptors located on GABAergic interneurons in the PFC; this mechanism of action leads to disinhibition of prefrontal pyramidal cells and eventually to increased synaptogenesis (Gerhard et al., 2016). Specifically, ketamine acts onto NMDA receptors located on somatostatin- and PV-expressing neurons (Gerhard et al., 2020). Based on these findings, ketamine appears to be a valid pharmacological tool to manipulate prefrontal glutamatergic and GABAergic neurotransmissions and determine their contribution to emotional deregulations. However, the vast majority of studies on ketamine assessed the effects of the drug on the brain and behavior once changes in emotional behaviors were already established in rodents, precluding any potential interpretation in terms of resilience to stress. To address this issue, more recent works have used ketamine prophylactically (i.e., before environmental exposures known to induce changes in emotional behaviors—Amat et al., 2016; Brachman et al., 2016; McGowan et al., 2017; Mastrodonato et al., 2018). A single dose of ketamine given before stress prevents the development of depressive-like behaviors in male rodents. Unfortunately, these studies have not included assessments of the prefrontal glutamatergic and GABAergic transmission as potential modulators of ketamine-induced stress resilience. More importantly, they focused only on male subjects. This is rather surprising since sex-specific effects of ketamine when given after a stressful event have been reported (Franceschelli et al., 2015; Sarkar and Kabbaj, 2016). The goal of the present work was to address these limitations to enhance our understanding of potential sex-specific molecular mechanisms regulating resilience to stress-related mood disorders. We utilized a chronic stress paradigm in male and female mice to determine the extent to which prophylactic ketamine prevents changes in emotional behaviors and impacts the expression of genes that regulate NMDA receptor and PV-dependent GABAergic transmission in the PFC.

## Materials and Methods

### Subjects

Adult (8 weeks) C57Bl/6 male and female mice were used (Jackson Laboratory, Bar Harbor, ME, USA). Mice were group-housed per sex in our facility (four to five mice/cage unless specified otherwise) and maintained on a 12-h reverse light-dark cycle with food and water *ad libitum*. Animals were allowed 1 week of habituation to our facility before beginning experiments. All procedures were approved by the Office of Responsible Research Practices of the researchers’ institution and conformed to the U.S. National Institutes of Health Guide for the Care and Use of Laboratory Animals.

### Drug Treatment

Mice received a single intraperitoneal (i.p.) injection of vehicle (0.9% saline) or ketamine (10 mg/kg) 7 days before the beginning of the chronic stress period. This dose was chosen based on previous work showing that it is sufficient to decrease immobility behaviors in male and female mice in the forced swim test (FST) up to 24 h after the injection (Franceschelli et al., 2015; Gerhard et al., 2020). Because the effects of ketamine in females have been reported to vary according to the stage of the estrus cycle (Dossat et al., 2018), we used a vaginal swab to determine the estrus cycle stage at the time of injection. Females in di-estrus and met-estrus were combined in one group, and females in estrus were kept in another group. No female in pro-estrus was found.

### Prefrontal Gene Expression and Behaviors Before Chronic Stress Exposure

**Figure 1:**
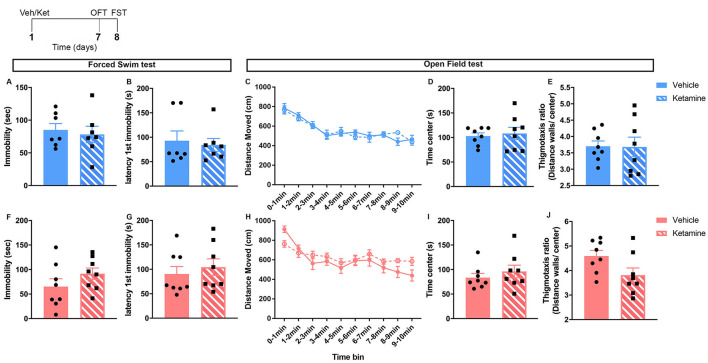
Behaviors in the forced swim and OFT were unchanged by ketamine 7 days after the injection in control male and female mice. Top left: schematic representation of the experimental timeline. Top row (blue; **A–E**): males; bottom row (pink; **F–J**): females. *N* = 8/group/sex. Data analyzed by the Student’s *t*-test. Veh, vehicle; Ket, ketamine; OFT, open field test; FST, forced swim test.

Seven days after injection with vehicle or ketamine, the first cohort of mice (*n* = 8 mice/sex/group; one video of the male FST was compromised leading to *n* = 7 for that group) was used to assess for sustained effects of ketamine on behaviors in the open field test (OFT) and FST in non-stressed mice (see Figure 1 for a schematic of experimental timeline; see below for a description of the behavioral assays).

**Figure 2:**
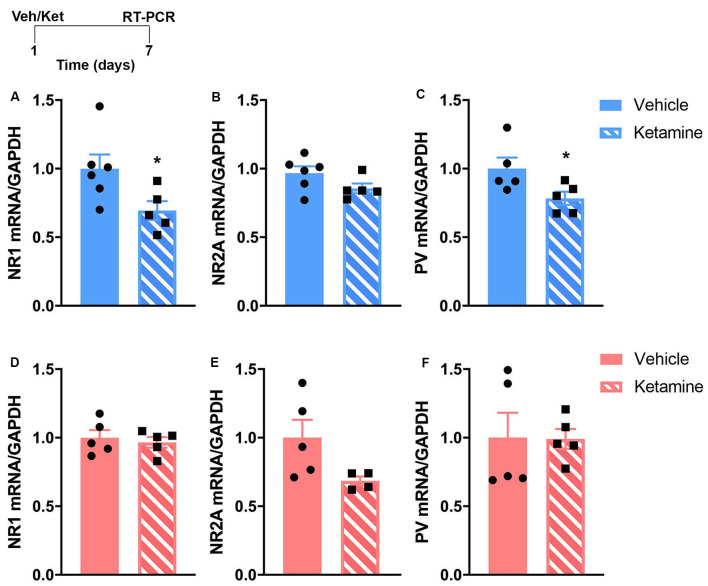
The sustained effects of ketamine exposure on mRNA levels of genes involved in glutamatergic and GABAergic transmission in the prefrontal cortex (PFC) are different in control male and female mice. Top left: schematic representation of the experimental timeline. Top row (blue; **A–C**): in males, mRNA levels of NR1 and parvalbumin (PV) are reduced 7 days post-ketamine injection. Bottom row (pink; **D–F**): in females, no changes are observed after ketamine injection at the same time point. *N* = 4–6 mice/group/sex. Data analyzed by the Student’s *t*-test. *p ≤ 0.05. Veh, vehicle; Ket, ketamine.

An additional cohort of mice (*n* = 5–6 mice/sex/group) was used to measure mRNA levels of genes regulating NMDA receptors (NR1 and NR2A subunits) and PV using RT-qPCR. The NR1 and NR2A subunits of the NMDA receptor were shown to modulate mice behaviors in the FST and OFT (Boyce-Rustay and Holmes, 2006; Halene et al., 2009). Our previous work showed that changes in PV mRNA levels correlate with levels of emotional behaviors in female mice after UCMS (Shepard et al., 2016) and that chemogenetic manipulation of prefrontal PV interneurons modulates levels of anxiety-like behaviors in the OFT (Page et al., 2019). Brains were collected 7-days post-injection (see Figure 2 for a schematic of the experimental timeline). The PFC was dissected in a cold room on dry ice following the instructions described by Spijker (2011). RNA was extracted from tissue using PureZOL RNA Isolation Reagent (Bio-Rad, Hercules, CA, USA) and NucleoSpin RNA II (Machery-Nagel, Allentown, PA, USA). cDNA templates were generated using the iScript Reverse Transcription kit (Bio-Rad, Hercules, CA, USA). The target cDNA and the reference target glyceraldehyde-3-phosphate dehydrogenase (GAPDH) were amplified simultaneously with SsoAdvanced SYBR Green Supermix in a CFX96 Real-Time PCR Detection System (Bio-Rad, Hercules CA, USA).

### Unpredictable Chronic Mild Stress (UCMS) Experimental Groups

**Figure 3:**
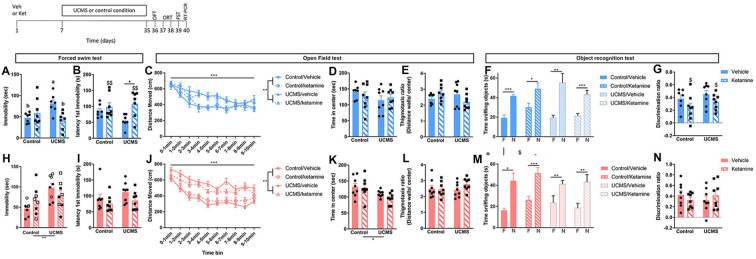
Prophylactic ketamine confers resilience to UCMS-induced changes in FST behaviors in males only. Top left: schematic representation of the experimental timeline. Top row (blue; **A–G**): in male mice, UCMS increases passive coping behaviors in the FST, which is prevented by prophylactic ketamine **(A,B)**. UCMS also increase general activity in the OFT, a phenotype that is not prevented by prophylactic ketamine **(C)**. Anxiety-like behaviors in the OFT are not affected by UCMS or ketamine **(D,E)**. While all the male mice show a preference for the novel object vs. the familiar one in the ORT, ketamine reduces the discrimination ratio **(F,G)**. Bottom row (pink; **H–N**): in female mice, UCMS-induced increased in passive coping strategy in the FST **(H,I)**, and anxiety-like behaviors and general activity in the OFT **(J–L)** are not prevented by prophylactic ketamine. In the FST **(H)**, the stage of the estrus cycle at the time of ketamine injection contributes to the effects (open-label: di- and met-estrus; closed label: estrus). Long-term memory measured in the object recognition test is not affected by UCMS or ketamine **(M,N)**. *N* = 7–8 mice/group/sex. Data analyzed by two-way ANOVA, with a repeated measure for locomotor activity per time bin, or by paired Student’s *t*-test to compare time spent sniffing the familiar object **(F)** vs. the new object **(N)**. *p ≤ 0.05, **p ≤ 0.01, ***p ≤ 0.001, ^a^ vs. ^b^*p* ≤ 0.05, ^$^main ketamine effect for *p* ≤ 0.05 or ^$$^*p* ≤ 0.01. Veh, vehicle; Ket, ketamine; UCMS, unpredictable chronic mild stress; OFT, open field test; ORT, object recognition test; FST, forced swim test.

The 4-week UCMS paradigm was shown to consistently and reliably induce changes in emotional behaviors in mice and are suitable to study the increased vulnerability of female subjects to stress-induced emotional dysregulation (Mineur et al., 2006; Nollet et al., 2013; Shepard et al., 2016; Shepard and Coutellier, 2018). UCMS mice are single-housed throughout the 4 weeks and exposed daily to alternating mild stressors presented in a random order according to an unpredictable schedule. Stressors included: the absence of nesting material for 24 h, 20° cage tilt on the vertical axis for 6 h, absence of bedding in the cage for 8 h, restraint stress in the dark for 8 min, and restraint stress under a bright light for 4 min. Control animals were group-housed and handled once daily for 1–2 min throughout the 4 weeks. Mice were divided into four groups according to a 2 × 2 experimental design, with control daily handling/UCMS and vehicle/ketamine as factors (*n* = 7–8 mice/group/sex). Twenty-four hours following the UCMS (or handling) period, mice were tested in the OFT, in the object recognition test (ORT), and in the FST (see below for a description of the behavioral assays). A subset of mice (*n* = 4–6/sex/group) was anesthetized 24 h after the FST and PFC collected to conduct RT-qPCR analyses as described above (see Figure 3 for a schematic of the experimental timeline).

### Behavioral Assessments

All behavioral tests were conducted during the dark phase of the light-dark cycle, and after a minimum of 1-h habituation to the testing room. For females, we recorded the stage of the estrus cycle by vaginal swab directly after each test. For the OFT, mice were placed individually in an unfamiliar square arena (40 × 40 cm) for 10 min and were free to explore. Behaviors were recorded using an overhead camera, and videos were scored offline using the Ethovision XT program (Noldus, The Netherlands). The total distance traveled (cm) was used as a measure of locomotor activity, while the time spent in the center of the arena, and the ratio of distance traveled near the walls vs. in the center (thigmotaxis ratio) were used as an indicator of anxiety-like behaviors.

The ORT was adapted from Ennaceur and Delacour (1988). The test took place 24 h after the OFT in the same arena. On the first day (learning session), two identical unfamiliar objects were placed equidistantly from the walls of the arena. Mice were allowed to explore the objects for 10 min. After a 24-h inter-trial interval (testing session), mice were placed back in the same arena with one object encountered during the learning session (now a familiar object) and with a novel unfamiliar object. Mice were again allowed to explore both objects over 10 min. Each session was recorded using an overhead camera and videos were scored off-line by an experimenter blind to the sex or group of the subjects. The time spent sniffing the objects during each session was scored and a discrimination ratio (DR) was calculated as an indicator of long-term memory (DR = time exploring unfamiliar object-time exploring familiar object/total time of object exploration).

The FST consisted of a single exposure to the test, similar to previous reports that studied the effects of ketamine on FST behaviors (Franceschelli et al., 2015; Ghosal et al., 2020). Briefly, mice were placed individually in a glass cylinder (height: 30 cm; diameter: 15 cm) filled with water (24–25°C) for 6 min. At the end of this period, mice were placed in a clean cage positioned on a heating pad to prevent hypothermia. Behaviors were recorded using a side camera and videos were scored off-line by an experimenter blind to the sex or group of the subjects. The latency to first immobility and the time spent immobile (in seconds) during the last 4 min of the test were used to assess for changes in despair-like behaviors and coping strategy to an acute swim stressor (Molendijk and de Kloet, 2019).

### Statistical Analyses

Data were analyzed using the software Prism 5.01 (GraphPad Software Inc., CA, USA). Male and female data were analyzed separately to assess the effect of chronic stress exposure and/or of ketamine in each sex. Data were analyzed using unpaired *t*-tests for the first group of mice, and using two-way ANOVAs followed by Tukey’s multiple comparison tests when appropriate for the UCMS group of mice. Locomotion in the OFT was analyzed by time bin using repeated measure ANOVAs. Time spent sniffing the novel vs. familiar object in the ORT was analyzed using a paired *t*-test. Because our previous work showed that high level of prefrontal PV mRNA could be associated with vulnerability to stress-related emotionality (Shepard et al., 2016), we conducted a correlation analysis (Pearson coefficient) between PV mRNA levels at the end of the chronic stress and behavioral testing period, and time spent immobile in the FST (the only behavioral endpoint showing an effect of ketamine after UCMS). In an attempt to obtain information about the potential contribution of the estrus cycle on the behavioral effects of ketamine in females, we included the stage of the estrus cycle (as high—di- and met-estrus—or low—estrus—levels of estradiol) at the time of ketamine injection and at the time of each behavioral assay as a covariate in our statistical model. The eight females per group divided equally into the two estrus stage groups leading to small sample size for each estrus stage group, Data are presented as mean ± standard error of the mean (SEM). Statistical significance was set at *p* ≤ 0.05.

## Results

### Distinct Sustained Molecular Changes in the PFC Induced by Ketamine in Male and Female Unstressed Mice Without Behavioral Changes

No major changes in behaviors were observed 7 days of post-ketamine injection in non-stressed mice (Figure 1). In both males and females, behavior in the FST was unchanged (Figures 1A,B,F,G). No ketamine effect was found in the open field behavior of males (Figures 1C–E). In females, time in center, thigmotaxis ratio, and locomotion were not changed (Figures 1H–J).

We examined gene expression in the PFC 7-days after ketamine injection using RT-qPCR. This time-point is equivalent to when our behavioral cohorts would have started stress exposure. Based on the known influence of ketamine on glutamatergic and GABAergic transmission (Gerhard et al., 2016, 2020), and on our previous work showing that UCMS caused changes in PV expression in the PFC of male and female mice that correlated with changes in emotional behavior (Shepard et al., 2016), we hypothesize that prophylactic ketamine provides resilience to stress-induced changes in emotional deregulations *via* alterations in excitation and inhibition in the PFC. Specifically, based on our published work (Shepard et al., 2016; Page et al., 2019), we hypothesized that decreased level of PV mRNA will be associated with resilience to stress-induced changes in emotional behaviors. We quantified changes in mRNA levels of NMDA receptor subunits and PV in response to ketamine. The RT-qPCR analysis revealed that males experienced more changes in the expression of prefrontal markers of NMDA receptors and PV-dependent GABAergic transmission. mRNA levels of NR1 and PV were significantly reduced in the PFC of males 7 days post-ketamine (Figures 2A,C; NR1: *t*_(9)_ = 2.35, *p* = 0.04; PV: *t*_(8)_ = 2.30, *p* = 0.05). In females, no significant change was found (Figures 2D–F). However, the trending ketamine-induced decrease in NR2A mRNA levels (*p* = 0.07; Figure 2E) should be further investigated due to our small sample size (*n* = 4/5 per group).

### Prophylactic Ketamine Induces Resilience to Chronic Stress-Induced Behavioral Changes in the FST in Males, but Not Females

The goal was to assess whether a single dose of prophylactic ketamine could prevent the development of chronic stress syndrome in male and female mice. After 4 weeks of UCMS, we exposed mice to a series of behavioral tests. In males, UCMS-induced increased in immobility in the FST was prevented by the pre-treatment with ketamine (Figure 3A; significant interaction between UCMS and ketamine: *F*_(1,25)_ = 8.38, *p* = 0.008; *post hoc* analyses: UCMS/vehicle vs. control/vehicle *p* = 0.05 and vs. UCMS/ketamine *p* = 0.02). Prophylactic ketamine also delayed the latency to first immobility (Figure 3B; *F*_(1,25)_ = 8.46, *p* = 0.008), especially after exposure to UCMS (UCMS/vehicle vs. UCMS/ketamine *p* = 0.02). In the OFT, UCMS induced hyperactivity (main UCMS effect *F*_(1)_ = 11.085, *p* = 0.003; main time bin effect *F*_(9)_ = 25.69, *p* < 0.0001) that was not prevented by ketamine (Figure 3C). UCMS did not affect time spent in the center (Figure 3D), as previously reported in males (Shepard et al., 2016), while a significant interaction between UCMS and ketamine was found for the thigmotaxis ratio (Figure 3E; *F*_(1,27)_ = 4.53, *p* = 0.03) but *post hoc* analyses did not reveal significant differences between groups.

In females, UCMS increased immobility in the FST (Figure 3H; main UCMS effect *F*_(1,28)_ = 10.17, *p* = 0.004). However, unlike males, prophylactic ketamine did not prevent this UCMS effect (no main or interaction effect of ketamine). Interestingly, we observed a trend for an effect of the estrus cycle stage at the time of the ketamine injection (*F*_(1,28)_ = 3.07, *p* = 0.09), whereby females injected with ketamine in di- or met-estrus have higher immobility time than females injected with ketamine when in estrus. No change in latency to first immobility was observed (Figure 3I), and the stage of the estrus cycle at the time of the FST did not affect results. Similar to males, females exposed to UCMS showed hyperactivity (main effect of UCMS *F*_(1)_ = 69.43, *p* < 0.0001; main time bin effect *F*_(9)_ = 28.53, *p* < 0.0001), which was not prevented by ketamine (Figure 3J). Finally, while the thigmotaxis ratio was not affected by UCMS or ketamine (Figure 3L), UCMS reduced center time (Figure 3K; *F*_(1,28)_ = 6.78; *p* = 0.015), which was not prevented by ketamine. No effect of the estrus cycle stage at the time of the ketamine injection or at the time of testing was found for the behaviors measured in the OFT.

Because ketamine use has been associated with cognitive deficits, we assessed long-term memory in mice using the ORT. Males and females of all groups showed a preference for the novel object over the familiar one during the testing phase of the assay (Figures 3F,M). However, we observed that a single dose of ketamine reduced the discrimination ratio in males, independently of UCMS exposure (Figure 3G; main effect of ketamine: *F*_(1,27)_ = 4.89; *p* = 0.03).

### Ketamine Interacts With Chronic Stress to Reduce mRNA Levels of Parvalbumin in the PFC of Males, Which Correlates With Lower Time Spent Immobile in the FST

**Figure 4:**
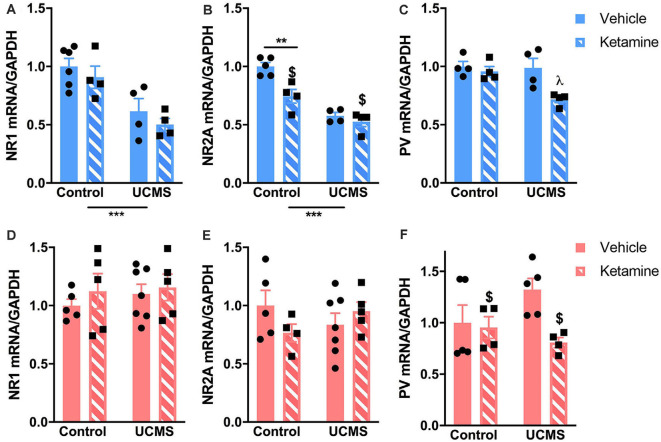
Ketamine and UCMS interact to change mRNA levels of genes involved in glutamatergic and GABAergic transmission in the PFC. Top row (blue; **A–C**): in males, mRNA levels of NR1 and NR2A are significantly reduced by UCMS. NR2A mRNA levels are also reduced by ketamine, mostly in the control group. PV mRNA levels are significantly lower in the PFC of mice exposed to UCMS and ketamine. Bottom row (pink; **D–F**): in females, only PV mRNA levels were reduced by ketamine. *N* = 4–6 mice/group/sex. Data analyzed by two-way ANOVA. **p ≤ 0.01, ***p ≤ 0.001, ^$^main ketamine effect for *p* ≤ 0.05, ^λ^different than all other groups for *p* ≤ 0.03.

We measured mRNA levels of markers of NMDA receptor subunits and PV-dependent GABAergic transmission in the PFC of mice exposed to UCMS after vehicle or ketamine treatment. In males, UCMS decreased levels of NR1 (Figure 4A; *F*_(1,14)_ = 22.29, *p* = 0.0003) and NR2A (Figure 4B; *F*_(1,13)_ = 57.6, *p* < 0.0001). By comparison, no UCMS effects were found in females for NR1 or NR2A (Figures 4D,E). Ketamine significantly reduced mRNA levels of NR2A in males (*F*_(1,13)_ = 13.19, *p* = 0.003), an effect mostly driven by a reduction of NR2A mRNA in control mice injected with ketamine vs. vehicle (significant interaction *F*_(1,13)_ = 5.97, *p* = 0.03; *post hoc* test: control/vehicle vs. control/ketamine *p* = 0.03). Ketamine also reduced mRNA levels of PV in both males and females (Figures 4C,F; males: *F*_(1,12)_ = 8.87, *p* = 0.01; females: *F*_(1,14)_ = 4.89, *p* = 0.04). Importantly, UCMS interacted with ketamine to reduce PV mRNA levels especially in males (interaction: *F*_(1,12)_ = 4.75, *p* = 0.01; *post hoc* analyses: UCMS/ketamine < all other groups for *p* < 0.03). In females, the main ketamine effect is mostly driven by the reduced level of PV mRNA in the UCMS group after the ketamine treatment (interaction: *F*_(1,14)_ = 3.42, *p* = 0.08; *post hoc* analyses: UCMS/ketamine < UCMS/vehicle for *p* = 0.053). The correlation analysis between PV mRNA at the end of the testing period and the immobility time in the FST showed that in males low levels of PV mRNA in the PFC correlates with low levels of immobility in the FST (Pearson’s coefficient = 0.472; *p* = 0.038), while the same was not true in females (Pearson’s coefficient = 0.295; *p* = 0.117—see Supplementary Figure 1).

## Discussion

The findings of the present study reveal that a low dose of ketamine, when used as a prophylactic drug, promotes resilience to some behavioral changes induced by chronic stress exposure in male mice, but fails to confer such protection in females. They also show that ketamine has sustained effects on the expression of genes that regulate glutamatergic and GABAergic transmission in the PFC and that these effects differ in males and females. While a causal relationship was not established here between gene expression changes and behavioral resilience to stress, our results support the idea that the state of the glutamatergic/GABAergic circuits in the PFC before exposure to chronic stress might directly contribute to resilience to stress-related mood disorders and that further studying sex differences in ketamine effects on those circuits might help our understanding of the mechanisms underlying increased vulnerability to emotional deregulations in females.

Based on our model of chronic stress, our behavioral data support previous research showing that ketamine exposure prevents the stress-induced increase in despair-like behaviors in the FST in male mice (Brachman et al., 2016; Mastrodonato et al., 2018). While immobility in the FST is traditionally interpreted as a measure of “depressive-like behaviors,” recent criticisms of the test (Reardon, 2019) have led to the conclusion that it is rather an approach to assess an animal strategy to cope with acute inescapable stress (Molendijk and de Kloet, 2019). In this sense, an active coping style (analogous to less immobility in the FST) is characteristic of individuals with low hypothalamic pituitary adrenal (HPA) axis activity and higher resilience to specific stressors (Veenema et al., 2003; Wisłowska-Stanek et al., 2019). Our data, therefore, support that prophylactic ketamine increases resilience to stress as shown by lower immobility time in the FST. However, prophylactic ketamine does not provide resilience against chronic stress-induced hyperactivity tested in the OFT. This observation replicates previous findings (Brachman et al., 2016) showing that only a subset of behavioral domains that are sensitive to stress exposure are also sensitive to ketamine treatment. It is unlikely that changes in behaviors in the FST are due to the increase in locomotor activity observed following chronic stress exposure. Increased locomotion following chronic stress has been reported by others (e.g., Boulle et al., 2014). If anything, increased locomotor activity would have resulted in a higher level of active behaviors in the FST and increased total sniffing time in the ORT, which was not the case here.

In females, none of the chronic stress-induced changes in behaviors were prevented by pre-exposure to ketamine. Similar to males, UCMS-induced changes in behaviors in the OFT (elevated anxiety-like behaviors and hyperactivity) remained despite pre-treatment with ketamine. Importantly, the increase in despair-like behavior in the FST after chronic stress exposure was still observed in females that received prophylactic ketamine. The absence of a ketamine effect on this behavior is surprising considering that previous work in rodents showed heightened sensitivity of females to ketamine given after stress exposure (Franceschelli et al., 2015; Sarkar and Kabbaj, 2016), and given Dolzani’s data showing resilience to the effects of inescapable tail shock in female rats after prophylactic ketamine (Dolzani et al., 2018). However, unlike Dolzani’s protocol, we used a long chronic stress procedure that extents to 5 weeks after the ketamine injection (vs. 1 week in Dolzani’s study). Differences in species and dose could also explain the discrepancies in results. Studies show that while both male and female mice experience a reduction in immobility in the FST 24 h after a single injection of ketamine (10 mg/kg; Gerhard et al., 2020), unlike males, female mice do not benefit from sustained antidepressant effects of ketamine (7-day post-treatment; Franceschelli et al., 2015). Reports from human studies support the idea that ketamine effects last longer in males (Coyle and Laws, 2015; Rybakowski et al., 2017). This suggests that by the time female mice were facing the bulk of our chronic stressors, ketamine effects on pathways that could induce resilience were already severely reduced, if not completely absent. This is supported by our gene expression analysis conducted 7-days post-ketamine injection showing no difference between vehicle-injected and ketamine-injected females. It is also important to note that the stage of the estrus cycle at the time of the ketamine injection could play a role in our findings. Others reported that females in pro-estrus have increased sensitivity to the anti-depressant effects of a low dose of ketamine (Dossat et al., 2018). Here, none of our females were in pro-estrus at the time of injection; but our findings suggest similarly that the prophylactic effects of ketamine to prevent the development of depressive-like behavior in the FST might be modulated by estradiol levels at the time of injection. While our interpretation of our analysis of the estrus cycle at the time of injection is limited by the small sample size, our data are informative especially as they support other recent findings showing that ovarian hormones are necessary for prophylactic ketamine to drive resilience to stress in female mice (Chen et al., 2020). This adds to the current knowledge that gonadal hormones mediate the effects of ketamine on emotional behaviors. This topic deserves further investigation, including a design that is sufficiently powered to address directly the role of the estrus cycle stage on ketamine effects on emotional behaviors in females. This is of particular interest considering the higher rate of depression in females, and the current lack of efficient and efficacious treatments.

The sex-specific effects of ketamine in conferring resilience to stress-induced increased in emotional behaviors are paralleled by differences in ketamine-induced changes in the prefrontal glutamatergic and PV systems. We focused our gene expression analysis on markers of NMDA receptor and PV-dependent GABAergic transmissions since previous work showed that increased PV cells activity in the PFC, potentially due to increased glutamatergic input, could contribute to increased anxiety-like behaviors after stress in females but not males (Page et al., 2019). Furthermore, preclinical and clinical findings suggest that ketamine acts on NMDA receptors located on inhibitory neurons, including somatostatin and PV interneurons, thereby leading to disinhibition of PFC pyramidal cells and a burst of glutamate that contributes to the sustained effects of ketamine through synaptic and spine plasticity (Duman and Aghajanian, 2012; Stone et al., 2012; Moda-Sava et al., 2019; Gerhard et al., 2020). Our data show that a single ketamine injection induces sustained changes in male prefrontal expression of genes regulating NMDA receptors and PV that could contribute to their resilience to chronic stress. At the time chronic stress was initiated (1 week after the ketamine injection), prefrontal expression levels of NR1 and PV were downregulated, while levels of NR2A are unchanged. Previous reports indicate that the anti-depressant effects of ketamine are independent of the NR2A subunit of the NMDA receptor (Gerhard et al., 2020). Ketamine effects on NR1 and PV have been more studied. Others reported decreases in NR1 and PV expression lasting up to 2 days after an acute injection of ketamine (Tang et al., 2015; Zhou et al., 2015). The difference in the length of the ketamine effects between these studies and our findings can reflect differences in animals (mice vs. rats) and doses (30 mg/kg vs. 10 mg/kg). While our approach does not allow us to determine whether the decrease in NR1 expression is specific to a cell type in the PFC, it is tempting to suggest that the stress-resilient effects of ketamine are driven by reduced expression of NR1 on PV-interneurons. In support of this idea, we previously showed that the chronic stress-induced increased in emotional behaviors in mice is associated with an increased number of glutamatergic terminals onto prefrontal PV neurons (Shepard and Coutellier, 2018). Others showed that the anti-depressant effect of ketamine is blocked in mice with reduced levels of NMDA receptor on prefrontal GABA interneurons (somatostatin- and parvalbumin-expressing neurons) but not glutamate neurons (Wohleb et al., 2017; Gerhard et al., 2020). Whether a similar mechanism drives the prophylactic effects of ketamine would need to be determined, especially since others provided evidence that the mechanisms underlying the anti-depressant vs. prophylactic behavioral effects of ketamine are different in the hippocampus (LaGamma et al., 2018). Cell type-specific transgenic mouse models or single-cell RNA sequencing approaches could help address this question.

The reduced level of PV expression 7 days post-ketamine injection would support decreased sensitivity of these cells to glutamatergic transmission since PV expression is activity-dependent and responds to glutamatergic input (Caballero and Tseng, 2016). Others reported a reduced number of PV neurons in the PFC 30 min after ketamine injection in male mice (10 mg/kg) and associated these changes at the cell level with the psychotomimetic effects of ketamine (Yang et al., 2015). We do not observe changes in locomotor activity 1- or 5-weeks post-ketamine in non-stressed mice. A sustained reduced sensitivity of PV-interneurons to glutamate could offset the effects of chronic stress-induced enhancement in glutamatergic transmission on this neuronal population (Farley et al., 2012; Shepard and Coutellier, 2018), potentially preventing their over-active phenotype (Page et al., 2019) and provide resilience to stress-induced changes in emotional behaviors. This hypothesis needs to be verified using more direct methodologies, including for instance specific ablation of NR1 from PV cells through genetic approaches. It is important to observe that in non-stressed animals, ketamine injection does not lead to change in behaviors at the 1-week and 5-week post-injection time points. The fact that changes in NR1 and PV mRNA levels 1-week post-injection does not correlate with changes in emotional behaviors is however not surprising and replicate previous studies showing that the behavioral effects of ketamine are specific to stress-induced behaviors (e.g., Amat et al., 2016; Brachman et al., 2016; Dolzani et al., 2018). Other systems than the prefrontal glutamatergic/GABAergic ones are likely impacted by stress and interact with each other to drive changes in behaviors.

Interestingly, the sole long-lasting (5 weeks post-injection) effect of ketamine we observed in non-stressed mice is the reduced performance in the long-term memory task. This cognitive impairment 5 weeks after the ketamine injection in male mice is associated with a decreased level of the NR2A mRNA in non-stressed mice, while both NR1 and PV mRNA returned to vehicle-injected mice value at that same time point. Return to baseline levels 5 weeks post-ketamine injection was anticipated since ketamine seems to mostly impact synaptogenesis, and not glutamatergic/GABAergic transmission, on the long-term in the PFC (Moda-Sava et al., 2019). The long-lasting decrease in NR2A mRNA levels in both control and stressed mice is intriguing and could contribute to the cognitive deficits observed. The NR2A subunit of the NMDA receptor is necessary for long-term potentiation and synaptic plasticity in the cortex, particularly in the anterior cingulate cortex, that are important to form long-term memory (Massey et al., 2004; Toyoda et al., 2006). To our knowledge, no other work studied the effects of ketamine on NMDA receptor subunits 5 weeks post its injection in adults; further work is needed to validate our observation of long-lasting reduction of prefrontal NR2A mRNA levels, which can have important medical implication for the use of ketamine for the treatment of depression.

In females, ketamine has different effects on prefrontal genes that regulate the expression of NMDA receptor subunits and PV: no changes in expression of NR1, NR2A, and PV were observed 7-days post-ketamine injection in females. The absence of changes in the expression of our targeted genes in the female PFC could explain in part the absence of changes in behaviors before and after chronic stress. However, we cannot exclude that ketamine has a sustained effect on other prefrontal genes expression or might exert its resiliency effect through non-prefrontal mechanisms, including synaptic plasticity in the hippocampus (Mastrodonato et al., 2018; Krzystyniak et al., 2019). More in-depth analysis of changes in gene expression in the PFC and other brain regions of males and females is needed, and causal relationships between transcriptional and behavioral changes have to be established.

Finally, our results showed that ketamine interacts with chronic stress to modulate PV expression in the PFC of both male and female mice. Our previous work demonstrated that high prefrontal PV expression and increased activity of PV neurons directly contribute to a stress-induced increase in emotional behaviors (Shepard et al., 2016; Page et al., 2019). We would therefore expect that lower PV mRNA levels in the PFC of mice lead to dampened stress effects on emotional behaviors. Males show this association with resilience to stress-induced changes in the FST, but females still display high levels of passive coping behaviors and anxiety-like behaviors. This finding supports partly our hypothesis as we validated this correlation between PV mRNA and emotional behaviors only in males, while our previous work demonstrated a stronger association in females (Shepard et al., 2016). Interestingly, PV mRNA levels were reduced by ketamine *before* and after stress only in males, which could suggest that PV might contribute to stress resilience if its expression is reduced as early as the beginning of stress exposure, and throughout the entire period of stress. On the contrary, decreased PV expression *after the* onset of stress (as observed in females) might not be sufficient to induce a resilient phenotype. This idea is supported by some of our unpublished data showing that decreasing the activity of prefrontal PV neurons with chemogenetics is insufficient to restore normal emotional behaviors in both male and female mice after chronic stress exposure. Manipulation of activity of PV neurons starting at the beginning of the stress period will help us confirm that early decrease in their activity (and therefore decreased PV expression) is the key to resilience to stress-induced increase in emotional behaviors. So far, the timing of decreased PV mRNA levels of females exposed to UCMS and ketamine is unknown and is likely to be further complicated by gonadal hormones (Carrier and Kabbaj, 2013). Whether variability in the estrus cycle in our cohort of females influences ketamine- and UCMS-induced changes in PV mRNA levels needs to be determined. As a whole, the dynamic of changes in PV expression in response to ketamine remains unclear. Here we showed that under stressful conditions, PV expression is reduced up to 5 weeks post-ketamine injection, while others showed that up to 8 h after the injection PV neurons display enhanced activity under acute stress conditions (Ng et al., 2018). The dynamic response of PV interneurons to ketamine deserves more research, especially since it appears to be sex-specific and since it contributes to the therapeutic efficacy of ketamine (Gerhard et al., 2020).

In conclusion, our findings replicate previous works showing that prophylactic ketamine induces some resilience to stress in male rodents and that this effect might potentially be due to changes in PV-dependent GABAergic neurotransmission within the PFC. For the first time, our study included the variable of sex, which is of particular importance considering that female subjects are more susceptible to the effects of stress on mood and affective behaviors, as demonstrated here by the appearance of an anxiety-like phenotype in addition to the depressive-like phenotype after UCMS. We highlight that the prefrontal mechanisms underlying stress resiliency in males and females might be different. This novel finding needs to be further investigated to obtain sufficient preclinical information that could eventually lead to sex-specific clinical solutions to prevent the development of stress-related mood disorders.

## Data Availability Statement

The raw data supporting the conclusions of this article will be made available by the authors, without undue reservation.

## Ethics Statement

The animal study was reviewed and approved by Office of Responsible Research Practices of the Ohio State University.

## Author Contributions

TO and RS conducted the UCMS experiment and analyzed the mice behaviors. RS and EL conducted the short-term effects of ketamine behavioral experiments. EL conducted the RT-PCR analyses. LC designed the experiments, analyzed the data, and wrote the manuscript. All authors contributed to the article and approved the submitted version.

## Conflict of Interest

The authors declare that the research was conducted in the absence of any commercial or financial relationships that could be construed as a potential conflict of interest.

## References

[B1] Amat J. Dolzani S. D. Tilden S. Christianson J. P. Kubala K. H. Bartholomay K.. (2016). Previous ketamine produces an enduring blockade of neurochemical and behavioral effects of uncontrollable stress. J. Neurosci. 36, 153–161. 10.1523/JNEUROSCI.3114-15.2016 26740657 PMC4701957

[B2] Bangasser D. A. Valentino R. J. (2014). Sex differences in stress-related psychiatric disorders: neurobiological perspectives. Front. Neuroendocrinol. 35, 303–319. 10.1016/j.yfrne.2014.03.008 24726661 PMC4087049

[B3] Baxter L. R. Jr. Schwartz J. M. Phelps M. E. Mazziotta J. C. Guze B. H. Selin C.. (1989). Reduction of prefrontal cortex glucose metabolism common to three types of depression. Arch. Gen. Psychiatry 46, 243–250. 10.1001/archpsyc.1989.01810030049007 2784046

[B4] Berman R. M. Cappiello A. Anand A. Oren D. A. Heninger G. R. Charney D. S.. (2000). Antidepressant effects of ketamine in depressed patients. Biol. Psychiatry 47, 351–354. 10.1016/s0006-3223(99)00230-9 10686270

[B5] Boulle F. Massart R. Stragier E. Païzanis E. Zaidan L. Marday S.. (2014). Hippocampal and behavioral dysfunctions in a mouse model of environmental stress: normalization by agomelatine. Transl. Psychiatry 4:e485. 10.1038/tp.2014.125 25423137 PMC4259995

[B6] Boyce-Rustay J. M. Holmes A. (2006). Genetic inactivation of the NMDA receptor NR2A subunit has anxiolytic- and antidepressant-like effects in mice. Neuropsychopharmacology 31, 2405–2414. 10.1038/sj.npp.1301039 16482087

[B7] Brachman R. A. McGowan J. C. Perusini J. N. Lim S. C. Pham T. H. Faye C.. (2016). Ketamine as a prophylactic against stress-induced depressive-like behavior. Biol. Psychiatry 79, 776–786. 10.1016/j.biopsych.2015.04.022 26037911 PMC4633406

[B8] Caballero A. Tseng K. Y. (2016). GABAergic function as a limiting factor for prefrontal maturation during adolescence. Trends Neurosci. 39, 441–448. 10.1016/j.tins.2016.04.010 27233681 PMC4930717

[B9] Carrier N. Kabbaj M. (2013). Sex differences in the antidepressant-like effects of ketamine. Neuropharmacology 70, 27–34 10.1016/j.neuropharm.2012.12.009 23337256

[B10] Chen B. K. Luna V. M. LaGamma C. T. Xu X. Deng S. X. Suckow R. F.. (2020). Sex-specific neurobiological actions of prophylactic (R,S)-ketamine, (2R,6R)-hydroxynorketamine and (2S,6S)-hydroxynorketamine. Neuropsychopharmacology 45, 1545–1556. 10.1038/s41386-020-0714-z 32417852 PMC7360766

[B11] Covington H. E. III. Lobo M. K. Maze I. Vialou V. Hyman J. M. Zaman S.. (2010). Antidepressant effect of optogenetic stimulation of the medial prefrontal cortex. J. Neurosci. 30, 16082–16090. 10.1523/JNEUROSCI.1731-10.2010 21123555 PMC3004756

[B12] Coyle C. M. Laws K. R. (2015). The use of ketamine as an antidepressant: a systematic review and meta-analysis. Hum. Psychopharmacol. 30, 152–163. 10.1002/hup.2475 25847818

[B13] de Graaf R. Bijl R. V. Smit F. Vollebergh W. A. Spijker J. (2002). Risk factors for 12-month comorbidity of mood, anxiety and substance use disorders: findings from the Netherlands Mental Health Survey and Incidence Study. Am. J. Psychiatry 159, 620–629. 10.1176/appi.ajp.159.4.620 11925301

[B14] Dolzani S. D. Baratta M. V. Moss J. M. Leslie N. L. Tilden S. G. Sørensen A. T.. (2018). Inhibition of a descending prefrontal circuit prevents ketamine-induced stress resilience in females. eNeuro 5:ENEURO.0025–18.2018. 10.1523/ENEURO.0025-18.2018 29516036 PMC5839773

[B15] Dossat A. M. Wright K. N. Strong C. E. Kabbaj M. (2018). Behavioral and biochemical sensitivity to low doses of ketamine: influence of estrous cycle in C57BL/6 mice. Neuropharmacology 130, 30–41. 10.1016/j.neuropharm.2017.11.022 29175352 PMC5749639

[B16] Duman R. S. Aghajanian G. K. (2012). Synaptic dysfunction in depression: potential therapeutic targets. Science 338, 68–72. 10.1126/science.1222939 23042884 PMC4424898

[B17] Duman R. S. Monteggia L. M. (2006). A neurotrophic model for stress-related mood disorders. Biol. Psychiatry 59, 1116–1127. 10.1016/j.biopsych.2006.02.013 16631126

[B18] Ennaceur A. Delacour J. (1988). A new one-trial test for neurobiological studies of memory in rats. 1: behavioral data. Behav. Brain Res. 31, 47–59. 10.1016/0166-4328(88)90157-x 3228475

[B19] Farley S. Dumas S. El Mestikawy S. Giros B. (2012). Increased expression of the vesicular glutamate transporter-1 (VGLUT1) in the prefrontal cortex correlates with differential vulnerability to chronic stress in various mouse strains: effects of fluoxetine and MK-801. Neuropharmacology 62, 503–517 10.1016/j.neuropharm.2011.09.010 21945287

[B20] Franceschelli A. Sens J. Herchick S. Thelen C. Pitychoutis P. M. (2015). Sex differences in the rapid and the sustained antidepressant-like effects of ketamine in stress-naïve and “depressed” mice exposed to chronic mild stress. Neuroscience 290, 49–60. 10.1016/j.neuroscience.2015.01.008 25595985

[B21] Garrett J. E. Wellman C. L. (2009). Chronic stress effects on dendritic morphology in medial prefrontal cortex: sex differences and estrogen dependence. Neuroscience 162, 195–207. 10.1016/j.neuroscience.2009.04.057 19401219 PMC2720075

[B22] Gerhard D. M. Pothula S. Liu R. J. Wu M. Li X. Y. Girgenti M. J.. (2020). GABA interneurons are the cellular trigger for ketamine’s rapid antidepressant actions. J. Clin. Invest. 130, 1336–1349. 10.1172/JCI130808 31743111 PMC7269589

[B23] Gerhard D. M. Wohleb E. S. Duman R. S. (2016). Emerging treatment mechanisms for depression: focus on glutamate and synaptic plasticity. Drug Discov. Today 21, 454–464. 10.1016/j.drudis.2016.01.016 26854424 PMC4803609

[B24] Ghosal S. Duman C. H. Liu R. J. Wu M. Terwilliger R. Girgenti M. J.. (2020). Ketamine rapidly reverses stress-induced impairments in GABAergic transmission in the prefrontal cortex in male rodents. Neurobiol. Dis. 134:104669. 10.1016/j.nbd.2019.104669 31707118

[B25] Gold P. Chrousos G. P. (2002). Organization of the stress system and its dysregulation in melancholic and atypical depression: high vs. low CRH/NE states. Mol. Psychiatry 7, 254–275. 10.1038/sj.mp.4001032 11920153

[B26] Halene T. B. Ehrlichman R. S. Liang Y. Christian E. P. Jonak G. J. Gur T. L.. (2009). Assessment of NMDA receptor NR1 subunit hypofunction in mice as a model for schizophrenia. Genes Brain Behav. 8, 661–675. 10.1111/j.1601-183X.2009.00504.x 19563516 PMC2757454

[B27] Hodes G. E. Pfau M. L. Purushothaman I. Ahn H. F. Golden S. A. Christoffel D. J.. (2015). Sex differences in nucleus accumbens transcriptome profiles associated with susceptibility versus resilience to subchronic variable stress. J. Neurosci. 35, 16362–16376. 10.1523/JNEUROSCI.1392-15.2015 26674863 PMC4679819

[B28] Kendler K. S. (1998). Gender differences in the genetic epidemiology of major depression. J. Gend. Specif. Med. 1, 28–31. 11281009

[B29] Kessler R. C. (2003). Epidemiology of women and depression. J. Affect. Disord. 74, 5–13. 10.1016/s0165-0327(02)00426-3 12646294

[B30] Koike H. Iijima M. Chaki S. (2011). Involvement of AMPA receptor in both the rapid and sustained antidepressant-like effects of ketamine in animal models of depression. Behav. Brain Res. 224, 107–111. 10.1016/j.bbr.2011.05.035 21669235

[B31] Krzystyniak A. Baczynska E. Magnowska M. Antoniuk S. Roszkowska M. Zareba-Koziol M.. (2019). Prophylactic ketamine treatment promotes resilience to chronic stress and accelerates recovery: correlation with changes in synaptic plasticity in the CA3 subregion of the hippocampus. Int. J. Mol. Sci. 20:1726. 10.3390/ijms20071726 30965559 PMC6479955

[B32] LaGamma C. T. Tang W. W. Morgan A. A. McGowan J. C. Brachman R. A. Denny C. A. (2018). Antidepressant but not prophylactic ketamine administration alters calretinin and calbindin expression in the ventral hippocampus. Front. Mol. Neurosci. 11:404. 10.3389/fnmol.2018.00404 30459554 PMC6232342

[B33] Li N. Liu R. J. Dwyer J. M. Banasr M. Lee B. Son H.. (2011). Glutamate *N*-methyl-D-aspartate receptor antagonists rapidly reverse behavioral and synaptic deficits caused by chronic stress exposure. Biol. Psychiatry 69, 754–761. 10.1016/j.biopsych.2010.12.015 21292242 PMC3068225

[B34] Maeng S. Zarate C. A. Jr. Du J. Schloesser R. J. McCammon J. Chen G.. (2008). Cellular mechanisms underlying the antidepressant effects of ketamine: role of α-amino-3-hydroxy-5-methylisoxazole-4-propionic acid receptors. Biol. Psychiatry 63, 349–352 10.1016/j.biopsych.2007.05.028 17643398

[B35] Martinot J. L. Hardy P. Feline A. Huret J. D. Mazoyer B. Attar-Levy D.. (1990). Left prefrontal glucose hypometabolism in the depressed state: a confirmation. Am. J. Psychiatry 147, 1313–1317. 10.1176/ajp.147.10.1313 2399999

[B36] Massey P. V. Johnson B. E. Moult P. R. Auberson Y. P. Brown M. W. Molnar E.. (2004). Differential roles of NR2A and NR2B-containing NMDA receptors in cortical long-term potentiation and long-term depression. J. Neurosci. 24, 7821–7828. 10.1523/JNEUROSCI.1697-04.2004 15356193 PMC6729941

[B37] Mastrodonato A. Martinez R. Pavlova I. P. LaGamma C. T. Brachman R. A. Robison A. J.. (2018). Ventral CA3 activation mediates prophylactic ketamine efficacy against stress-induced depressive-like behavior. Biol. Psychiatry 84, 846–856. 10.1016/j.biopsych.2018.02.011 29615190 PMC6107435

[B38] Mayberg H. S. Lozano A. M. Voon V. McNeely H. E. Seminowicz D. Hamani C.. (2005). Deep brain stimulation for treatment-resistant depression. Neuron 45, 651–660. 10.1016/j.neuron.2005.02.014 15748841

[B39] McGowan J. C. LaGamma C. T. Lim S. C. Tsitsiklis M. Neria Y. Brachman R. A.. (2017). Prophylactic ketamine attenuates learned fear. Neuropsychopharmacology 42, 1577–1589. 10.1038/npp.2017.19 28128336 PMC5518899

[B40] Mineur Y. S. Belzung C. Crusio W. E. (2006). Effects of unpredictable chronic mild stress on anxiety and depression-like behavior in mice. Behav. Brain Res. 175, 43–50. 10.1016/j.bbr.2006.07.029 17023061

[B41] Moda-Sava R. N. Murdock M. H. Parekh P. K. Fetcho R. N. Huang B. S. Huynh T. N.. (2019). Sustained rescue of prefrontal circuit dysfunction by antidepressant-induced spine formation. Science 364:eaat8078. 10.1126/science.aat8078 30975859 PMC6785189

[B42] Molendijk M. L. de Kloet E. R. (2019). Coping with the forced swim stressor: current state-of-the-art. Behav. Brain Res. 364, 1–10. 10.1016/j.bbr.2019.02.005 30738104

[B43] Ng L. H. L. Huang Y. Han L. Chang R. C. Chan Y. S. Lai C. S. W. (2018). Ketamine and selective activation of parvalbumin interneurons inhibit stress-induced dendritic spine elimination. Transl. Psychiatry 8:272. 10.1038/s41398-018-0321-5 30531859 PMC6288154

[B44] Nollet M. Le Guisquet A. M. Belzung C. (2013). Models of depression: unpredictable chronic mild stress in mice. Curr. Protoc. Pharmacol. Chapter 5:Unit 5.65. 10.1002/0471141755.ph0565s61 23744712

[B45] Page C. E. Shepard R. Heslin K. Coutellier L. (2019). Prefrontal parvalbumin cells are sensitive to stress and mediate anxiety-related behaviors in female mice. Sci. Rep. 9:19772. 10.1038/s41598-019-56424-9 31875035 PMC6930291

[B46] Reardon S. (2019). Depression researchers rethink popular mouse swim tests. Nature 571, 456–457. 10.1038/d41586-019-02133-2 31337906

[B47] Rybakowski J. K. Permoda-Osip A. Bartkowska-Sniatkowska A. (2017). Ketamine augmentation rapidly improves depression scores in inpatients with treatment-resistant bipolar depression. Int. J. Psychiatry Clin. Pract. 21, 99–103. 10.1080/13651501.2017.1297834 28271731

[B48] Sarkar A. Kabbaj M. (2016). Sex differences in effects of ketamine on behavior, spine density, and synaptic proteins in socially isolated rats. Biol. Psychiatry 80, 448–456. 10.1016/j.biopsych.2015.12.025 26957131 PMC4940294

[B49] Schoevers R. A. Beekman A. T. Deeg D. J. Jonker C. van Tilburg W. (2003). Comorbidity and risk-patterns of depression, generalised anxiety disorder and mixed anxiety-depression in later life: results from the AMSTEL study. Int. J. Geriatr. Psychiatry 18, 994–1001. 10.1002/gps.1001 14618550

[B50] Shansky R. M. Hamo C. Hof P. R. Lou W. McEwen B. S. Morrison J. H. (2010). Estrogen promotes stress sensitivity in a prefrontal cortex-amygdala pathway. Cereb. Cortex 20, 2560–2657. 10.1093/cercor/bhq003 20139149 PMC2951843

[B51] Shepard R. Coutellier L. (2018). Changes in the prefrontal glutamatergic and parvalbumin systems of mice exposed to unpredictable chronic stress. Mol. Neurobiol. 55, 2591–2602. 10.1007/s12035-017-0528-0 28421533

[B52] Shepard R. Page C. E. Coutellier L. (2016). Sensitivity of the prefrontal GABAergic system to chronic stress in male and female mice: relevance for sex differences in stress-related disorders. Neuroscience 332, 1–12. 10.1016/j.neuroscience.2016.06.038 27365172

[B53] Shin L. M. Whalen P. J. Pitman R. K. Bush G. Macklin M. L. Lasko N. B.. (2001). An fMRI study of anterior cingulate function in posttraumatic stress disorder. Biol. Psychiatry 50, 932–942. 10.1016/s0006-3223(01)01215-x 11750889

[B54] Spijker S. (2011). “Dissection of rodent brain regions,” in Neuroproteomics, ed. Li K. W. (Totowa, NJ: Humana Press), 13–26.

[B55] Stone J. M. Dietrich C. Edden R. Mehta M. A. De Simoni S. Reed L. J.. (2012). Ketamine effects on brain GABA and glutamate levels with 1H-MRS: relationship to ketamine-induced psychopathology. Mol. Psychiatry 17, 664–665. 10.1038/mp.2011.171 22212598 PMC3883303

[B56] Tang J. Xue W. Xia B. Ren L. Tao W. Chen C.. (2015). Involvement of normalized NMDA receptor and mTOR-related signaling in rapid antidepressant effects of Yueju and ketamine on chronically stressed mice. Sci. Rep. 5:13573. 10.1038/srep13573 26315757 PMC4551989

[B57] Thompson S. M. Kallarackal A. J. Kvarta M. D. Van Dyke A. M. LeGates T. A. Cai X. (2015). An excitatory synapse hypothesis of depression. Trends Neurosci. 38, 279–294. 10.1016/j.tins.2015.03.003 25887240 PMC4417609

[B58] Toyoda H. Zhao M. G. Zhuo M. (2006). NMDA receptor-dependent long-term depression in the anterior cingulate cortex. Rev. Neurosci. 17, 403–413. 10.1515/revneuro.2006.17.4.403 17139841

[B59] Veenema A. H. Meijer O. C. de Kloet E. R. Koolhaas J. M. (2003). Genetic selection for coping style predicts stressor susceptibility. J. Neuroendocrinol. 15, 256–267. 10.1046/j.1365-2826.2003.00986.x 12588514

[B60] Wisłowska-Stanek A. Płaznik A. Kołosowska K. Skórzewska A. Turzynska D. Liguz-Lcznar M.. (2019). Differences in the dopaminergic reward system in rats that passively and actively behave in the Porsolt test. Behav. Brain Res. 359, 181–189. 10.1016/j.bbr.2018.10.027 30366032

[B61] Wohleb E. S. Gerhard D. Thomas A. Duman R. S. (2017). Molecular and cellular mechanisms of rapid-acting antidepressants ketamine and scopolamine. Curr. Neuropharmacol. 15, 11–20. 10.2174/1570159x14666160309114549 26955968 PMC5327447

[B62] Yang C. Shirayama Y. Zhang J. C. Ren Q. Yao W. Ma M.. (2015). R-ketamine: a rapid-onset and sustained antidepressant without psychotomimetic side effects. Transl. Psychiatry 5:e632. 10.1038/tp.2015.136 26327690 PMC5068814

[B63] Zarate C. A. Jr. Singh J. B. Carlson P. J. Brutsche N. E. Ameli R. Luckenbaugh D. A.. (2006). A randomized trial of an *N*-methyl-D-aspartate antagonist in treatment-resistant major depression. Arch. Gen. Psychiatry 63, 856–864. 10.1001/archpsyc.63.8.856 16894061

[B64] Zhou Z. Zhang G. Li X. Liu X. Wang N. Qiu L.. (2015). Loss of phenotype of parvalbumin interneurons in rat prefrontal cortex is involved in antidepressant- and propsychotic-like behaviors following acute and repeated ketamine administration. Mol. Neurobiol. 51, 808–819. 10.1007/s12035-014-8798-2 24973145

