# Supplementary material for: Sex Differences in the Sustained Effects of Ketamine on Resilience to Chronic Stress
Source: Front Behav Neurosci. 2020 Oct 20;14:581360. doi: 10.3389/fnbeh.2020.581360 (PMC7606988; doi:10.3389/fnbeh.2020.581360)
Supplement: Supplementary file 1 [file Data_Sheet_1.DOCX]

**Supplementary Data**


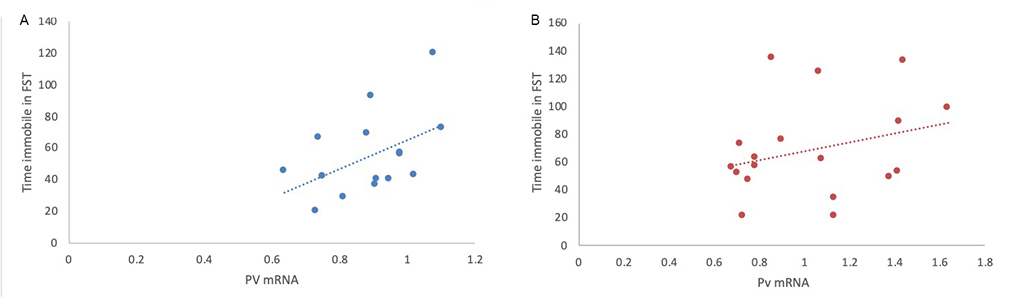


**Supplementary Figure 1:** Correlation analysis between level of PV mRNA in the PFC of male (A) and female (B) mice measured after 4 weeks of UCMS or control handling and Time spent immobile in the forced swim test (FST). Males: Pearson’s coefficient = 0.472; p=0.038; Females: Pearson’s coefficient = 0.295; p=0.117.
